# Pressure Ulcers from the Medico-Legal Perspective: A Case Report and Literature Review

**DOI:** 10.3390/healthcare10081426

**Published:** 2022-07-29

**Authors:** Filippo Gibelli, Paolo Bailo, Ascanio Sirignano, Giovanna Ricci

**Affiliations:** Section of Legal Medicine, School of Law, University of Camerino, 62032 Camerino, Italy; paolo.bailo@unicam.it (P.B.); ascanio.sirignano@unicam.it (A.S.); giovanna.ricci@unicam.it (G.R.)

**Keywords:** pressure ulcers, bedsores, medical liability

## Abstract

Introduction: The identification of professional liability profiles related to the development of pressure injuries is a very thorny issue from a medico-legal perspective. This is because no matter how strict the applied prevention protocols applied may be, the development of such injuries is largely dependent on endogenous factors. This paper aims to investigate the medico-legal issues related to this topic through the exposition of one case of medico-legal litigation and a traditional review of the literature. Methods: We performed a literature search using three databases (Pubmed, Scopus, and Web Of Science), restricting the search to the period between 2001 and 2021. We used “pressure ulcers” and “jurisprudence” as the main keywords. From an initial library of 236 articles, our selection resulted in 12 articles, which were included in the review. Results: We identified the ever-increasing expectations of patients and the concept of automatic attribution of responsibility when a pressure ulcer develops as the primary reasons for the increase in litigation over the past 20 years. The related corrective measures are numerous: a strict adherence to guidelines, an adequate documentation of preventive measures, a risk assessment, family involvement, and a successful collaboration between physicians and government institutions. Conclusions: The biological complexity of the pathogenetic development of pressure ulcers makes the subject very delicate from the medico-legal point of view. In principle, it is possible to state that a very large proportion of such injuries are preventable, but that there remains a percentage of them that cannot be prevented. In such cases, only a proper documentary demonstration of the adequacy of preventive measures can exclude liability profiles.

## 1. Introduction

Pressure ulcers (also known as decubitus ulcers, pressure injuries, or pressure sores) are defined as “localized damage to the skin and underlying soft tissue usually over a bony prominence or related to a medical or other device as a result of intense and/or prolonged pressure or pressure in combination with shear” [1]. These injuries most frequently localize in the adult population over the bony prominences of the hip and sacral region, but about 25% of cases are observed to involve the lower extremities [2]. Nevertheless, decubitus ulcers can be observed in the pediatric population, especially in the occipital region [3]. Pressure sores are generated precisely because of the pressure exerted on the tissues at a bony prominence that eventually leads to ischemia and necrosis [4]. In addition, friction [5] and excess moisture, which can cause skin maceration, are also recognized to play a role [6].

Pressure ulcers remain a significant problem on a global scale, despite the increased consideration and prevention of the phenomenon within healthcare facilities [7]. It is estimated that the prevalence of pressure ulcers in inpatients is around 5–15%, but in intensive care units and long-term care facilities it is significantly higher [2,8]. Although pressure ulcers can develop in any patient with skin subjected to pressure, we recognize categories of patients at increased risk who are elderly and/or persons with decreased mobility or sensitivity that are usually bedridden or wheelchair-bound [8,9].

Being affected by a pressure ulcer involves both serious medical complications and a significant reduction in the quality of life. In fact, the treatments to be carried out for the treatment of pressure ulcers are lengthy and substantially change one’s daily routine [10]. It is reported in the literature that living with a pressure ulcer involves pain, fear, anxiety, social isolation, and decreased independence [11,12]. From a medico-legal point of view, the development of pressure ulcers in a healthcare-assistance context can be of interest in ascertaining liability profiles for the healthcare workers involved. In this field, starting from the analysis of a case of health care liability related to the formation of pressure ulcers in a hospital context, we carry out a review of the current literature to determine whether these cases are identifiable as misconduct by health care professionals. In addition, we will also verify whether minimum requirements exist and are shared among the various healthcare facilities to prevent this phenomenon.

## 2. Case Report

**Figure 1:**
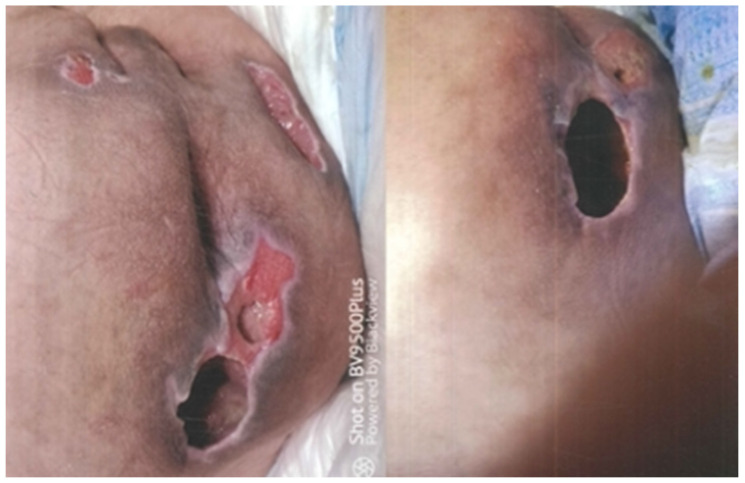
Situation at hospital discharge.

**Figure 2:**
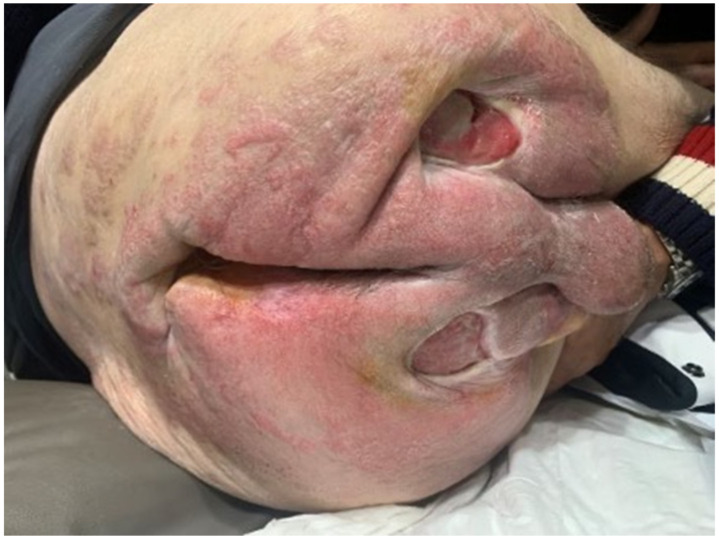
Situation Status one year after discharge from the hospital.

A 40-year-old man suffering from childhood encephalopathy with residual severe mental retardation and right brachio-crural hemiparesis was admitted to the neurosurgery department with a diagnosis of an expansive process in the posterior cranial fossa. A CT scan had in fact documented a gross hypodense expansive cerebellar formation compressing the fourth ventricle and that was responsible for the hydrocephalus. The patient underwent endoscopic ventriculocystotomy. During his subsequent stay in the hospital, he underwent two other surgical procedures, one to remove the cerebellar mass and the other to place an external spinal shunt. During his hospitalization, the patient was adequately followed from a neurosurgical and general medical point of view, but nowhere in the clinical diary was reference made to the presence of pressure ulcers nor, consequently, to their treatment. After discharge, the parents noted the presence of three voluminous pressure ulcers in the sacral and bilateral gluteal areas (Figure 1). The diagnostic and therapeutic procedure that characterized the evolution of the severe morbid condition was certainly adequate in terms of time and methods; however, in the medical record, there was never any reference to the presence and treatment of pressure sores, nor to those in the sacral region in particular. The patient made a claim for financial compensation for injuries sustained during his hospitalization. Figure 2 shows the clinical presentation one year after discharge from hospital. The event took place in 2021.

## 3. Materials and Methods

### 3.1. Searching Strategy

The research on the scientific literature was performed between January 2001 and December 2021 on the online databases of PubMed, Scopus, and Web of Science (WoS). The PubMed database search was performed by combining the Mesh terms “Pressure Ulcers” [Mesh] and “Jurisprudence” [Mesh] with the “AND” Boolean operator. We conducted the search on Scopus by entering the following search terms: “Pressure Ulcers”, “Bedsores”, “Pressure Sores”, and “Decubitus Ulcers” and combining them with the Boolean operator “OR”. Then, we performed a new search by inserting the following terms: “Legal Aspects”, “Court Decisions”, “Medical Jurisprudence”, “Litigation *”, and “Law *”, combining them with the operator “OR”. We set “title-abstract-keywords” as field tag. Finally, we combined the results of the two searches with the “AND” operator. The search on Web of Science was conducted similarly to the search on Scopus, the only difference being that we entered “title-abstract” as the tag field.

We performed a preliminary skimming independently: each author read the abstracts of the articles found and identified those they considered useful for the review. At the end of the preliminary evaluation procedure, the authors discussed the various articles, debating the suitability of the individual papers. At the end of the selection phase, the authors read all the articles to collect the data for the review.

### 3.2. Selection Criteria

The research initially provided 236 results. Specifically, 99 papers were found on PubMed, 85 on Scopus, and 52 on Web of Science. The types of the study objects of interest were the following: original articles, review articles, book chapters, conference papers, editorial materials, proceeding papers, and meeting abstracts. We conducted an initial pre-selection by removing duplicate papers (*n* = 24), articles not written in English (*n* = 12), and articles for which the full text was not available (*n* = 19). We then read the abstracts of the remaining 181 articles in order to identify papers suitable for reading the full text. At this stage, we decided to include only those articles that specifically addressed the medico-legal implications of pressure sores, excluding papers related to pressure sores in general terms, without reference to medical malpractice hypotheses. After reading the abstracts, we excluded 167 articles as not relevant to the purposes of the review.

Specifically, 24 papers were related to areas completely unrelated to the topic of interest (e.g., violence against the elderly and tube feeding of the elderly); 31 papers discussed general aspects of elderly care, without specific reference to pressure sores; 33 papers discussed general aspects of pressure sores, without analyzing the legal litigation issues (e.g., costs to the health care system); and 79 papers discussed how to prevent pressure sores, without any reference to the medical-legal implications. We then proceeded to read the full text of the remaining 14 papers, two of which were not included in the final review because they dealt with the medical-legal implications of pressure injuries only superficially, without providing useful elements for the purpose of the discussion. A total of 12 articles were included in the final review.

**Figure 3:**
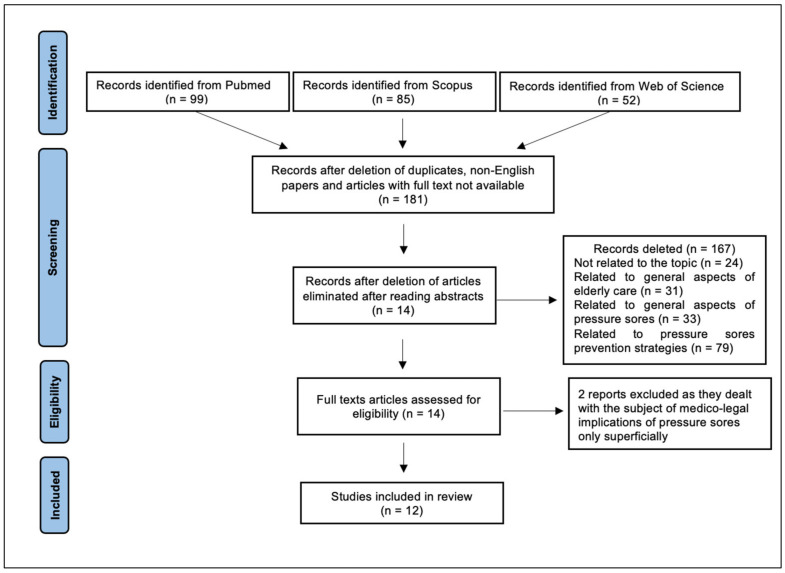
Review search strategy.

Figure 3 illustrates the article selection process.

### 3.3. Quality Evaluation

SANRA (Scale for the Assessment of Narrative Review Articles) [13] was employed for a quality check of the selected studies. The overall quality was determined as poor (score 0–6), moderate (7–9), or excellent (10–12). One article was found to be of poor quality, nine papers of moderate quality, and two articles of excellent quality. The results of SANRA are reported in Table 1.

### 3.4. Summary of Article Pool

The search identified 12 articles suitable for inclusion in this review. The 12 papers were published between 2002 and 2016. The socio-environmental and cultural background of the articles is mostly Anglo-American: 7 American papers, 3 English papers, 1 German paper, and 1 Australian paper. Regarding the types of articles, the vast majority (8) are perspective articles, in which the authors express their point of view regarding the reasons behind legal litigation regarding pressure sores in health care (both long-term and acute care) and formulate some operational proposals to reduce its scope. In addition, 2 review articles, 1 editorial, and 1 consensus paper were included in the review. Table 2 illustrates the main characteristics of the reviewed articles.

## 4. Results

The research has shown that since the 2000s there has been a substantial increase in litigation concerning pressure ulcers. This is because of the great progress in medical science, which has made it possible to achieve high standards of care also due to technological advances. However, this process of evolution has deployed sometimes dysfunctional effects, causing patients to have unrealistic expectations of health care [15,16,20,22]. This led to the development of the notion that any event, even a very remote and preventable event, is a source of liability if it results in harm to the patient. It is in fact based on this assumption that in many cases the realization of a pressure sore not present at admission to the hospital or the worsening of a pre-existing one is automatically interpreted as a result of malpractice [17,21,24]. The litigious spirit of patients or their relatives is then, in many cases, fed by judicial guidelines that increasingly tend to award damages for pressure injuries [19]. When documentary deficiencies are present; then, professional liability is almost automatic [14].

The authors of the reviewed papers propose several operational strategies aimed at improving the quality of care for pressure injuries and reducing litigation. One of the predominant strategies is undoubtedly the careful and painstaking documentation of all the measures that are taken by health care personnel to prevent the onset or worsening of pressure sores. References to guidelines should also be documented and failed attempts to implement measures should be noted [14,16,21]. It is also important that the guidelines are not applied uncritically and decontextualized, but that they are tailored to the individual case [17]. Probably the most relevant facet regarding the application of the guidelines should be the preliminary assessment of the risk of developing pressure injuries [18,24]. Another important point is training: it is crucial that all staff involved in the care process are adequately trained in pressure injury management and that they regularly update their training [17,21,23,25]. Some authors suggest that family involvement in the caregiving process could be beneficial, especially because of the creation of a cooperative relationship that would deter family members from filing a lawsuit [15]. Therefore, this highlights the importance of promoting a successful collaboration between physicians and institutions [19], in order to make the government bodies understand the centrality (also economic) of the problem of bedsores [20].

## 5. Discussion

The biological complexity of the pathogenetic mechanisms underlying the development of pressure ulcers makes the topic of a particular medico-legal interest. The core of the debate is in fact represented by the configurability of the elements of the preventability of the onset of injuries. In the genesis of decubitus lesions, external factors (pressure, shearing, friction, and moisture) exert their vulnerability more easily if they act on a subject with risk factors (immobility, sensory obnubilation, caloric-protein malnutrition, urinary and/or fecal incontinence, arterial and/or venous peripheral vasculopathy, hypoalbuminemia, edema, advanced age, sedation, dehydration, diabetes mellitus, anemia, arterial hypotension, and hyperthermia). Until the 2000s, there was a widespread belief that the onset of decubitus injuries was mostly to be considered an “inevitable” circumstance and independent from any profiles of medical malpractice, especially when the elderly and frail were affected. Gradually, the paradigm has undergone a substantial change, making decubitus injuries in fact interpretable in common opinion as an expression of deficiencies in care. This has naturally led to a substantial increase in litigation. Yet, upon closer inspection, the first evidence in the literature supporting the substantial preventability of pressure injuries dates to the 1980s. In 1988, Hibbs stated that pressure sores were preventable in 95% of cases [26]. This finding was later confirmed by numerous other authors, including Arblaster in 1999 [27], Clarkson in 2007 [21], and Stephens-Haynes in 2010 [28]. However, Hibbs’ finding was strongly criticized and questioned, and according to some authors, the percentage of truly preventable pressure sores (at least those of grade 3 and 4) would be less than half [29].

It is objectively very complex to determine what exact proportion of decubitus injuries are preventable, as the literature is discordant. What can be stated without fear of contradiction is that a proportion of pressure injuries are undoubtedly avoidable through appropriate preventive measures and monitoring. It is on the qualification of these measures that the medical-legal discussion should focus since the judicial disputes start from accusations of inadequacy regarding the adoption of measures of prevention and surveillance. The most appropriate prevention and monitoring paths possible must necessarily be codified by the guidelines developed by scientific societies and accredited organizations. This for two reasons: first, to ensure high-quality, highly effective clinical indications, and second, because the application of centrally validated guidelines makes it easier for healthcare professionals to demonstrate that they have correctly fulfilled their duty of care. There are essentially three documents internationally recognized as having the authoritative character of guidelines: the “Prevention and Treatment of Pressure Ulcers/Injuries: Clinical Practice Guideline” elaborated by the National Pressure Injury Advisory Panel (NPIAP), the European Pressure Ulcer Advisory Panel (EPUAP), and the Pan Pacific Pressure Injury Alliance (PPPIA) in 2009 and updated in 2014 and 2019; the “Consensus document: Role of dressings in pressure ulcer prevention”, issued by Wounds International in 2019; and the “White paper on the role of nutrition for pressure ulcer management” issued by the NPIAP, EPUAP, and PPPIA in 2015. There are also continent-wide guidelines: in the USA, the reference document is the “Guidelines on the prevention and management of pressure ulcers (injuries)” issued by the Wound, Ostomy, and Continence Nurses Society (WOCN) in 2016 [30]; in Europe, there are the already mentioned documents produced by NPIAP, EPUAP, and PPPIA; and in Asia, the main document is the “Guidelines for the Prevention and Management of Pressure Ulcers” of the Japanese Society of Pressure Ulcers, developed in 2005 and subsequently updated in 2009, 2012, and 2016 [31]. These are undoubtedly very useful tools for improving the management of injuries, identifying uniform and scientifically valid patterns of care and prevention, and containing legal disputes.

Regarding the reduction in medical-legal disputes, for the health care professional, having demonstrated that he or she has scrupulously adhered to the guidelines represents an element that places him or her relatively safe from the possibility of being held liable. However, it should be noted that the guidelines in general cannot be interpreted as tools to be applied uncritically or to all situations. In other words, there are many situations in which the particular conditions of the patient or health care facility dictate that the physician or nurse practitioner should not implement a particular guideline. This was an issue addressed in great depth by Hurwitz in 1998 [32], which makes the aspects of evaluating medical blame profiles in the context of decubitus injuries very complex. Beyond clinical and organizational situations that may make it imperative for physicians to deviate from guidelines, the actual applicability of complex preventive algorithms should be considered in healthcare settings that are often highly care-intensive. In fact, the aforementioned guidelines are not immediately applicable precisely because they are technically very valid and scientifically very thorough. In other words, the proper implementation of the provisions contained in the guidelines requires a significant amount of time and effort by the medical staff. This may not be particularly problematic in low-intensity care settings, but may, on the contrary, be impractical in set-tings with high patient flows, which are critical and requiring the application of numerous other therapeutic algorithms for other pathologies. The statement “*compliance with guidelines is equal to no health care liability*” therefore appears very simplistic and not in keeping with reality. A health care professional who has applied the guidelines to perfection can be found to be professionally liable because in the specific case there were reasons to deviate from them. Similarly, the liability of a health care professional who failed to implement the guidelines may be excluded because there were contingent conditions of an organizational nature that prevented him or her from implementing them.

Beyond these observations, the adherence to the guidelines is still a cornerstone of the policy for the management of pressure ulcers, as this adherence ensures the adequate documentation of the measures taken by medical team to prevent the onset or worsening of the wounds. As it is well known, the prevailing legal orientation is that the failure to document the adoption of a measure coincides with the failure to implement the measure itself. In other words, what is not documented has not been performed. One of the most important documentary elements is the risk assessment of developing pressure injuries. The three most used scales are the Braden Scale, the Norton Scale, and the Waterlow Scale. The Braden Scale, the most widely used in the US, consists of six items: sensory perception, moisture, activity, mobility, nutrition, and friction and shearing. The Norton Scale, developed in the UK, consists of five items: physical condition, mental condition, activity, mobility, and incontinence. The Waterlow Scale consists of nine items: build/weight in proportion to height, visual assessment of the skin in the area at risk, gender and age, continence, mobility, Malnutrition Screening Tool score, and special risk factors including tissue malnutrition, neurological deficit, and major surgery or trauma [33]. Risk scales are generally completed by nurses but are of great interest to physicians as well. In fact, the score of the scale, in addition to indicating the risk of the development of pressure sores, is also a predictor of mortality, the duration of hospitalization, and the type-of-care connotation of the patient.

With reference to the case reported, there was not even the slightest reference to preventive activities, and liability profiles in the civil law sphere have obviously been identified against the healthcare professionals of the hospital facility.

In the case presented, the decubitus lesions affected a common area, but it is particularly interesting to propose a concluding thought in relation to decubitus lesions occurring in completely unusual areas (such as the head). It is well known that the most common locations of pressure injuries are the sacrum, heels, trochanters, and elbows [34]. Therefore, it is legitimate to assume that the development of ulcers in infrequent regions may represent an additional obstacle for healthcare professionals to demonstrate the absence of liability profiles. This is because unusual areas are generally areas in which the pressure load is relatively low—less than the 32-mmHg is needed to overcome the pressure at the arteriolar end of the capillaries and compromise the microcirculation [35]. Therefore, the formation of ulcers in these areas would lead us to believe that an external pressure element, in addition to the weight of the body, has probably intervened. However, a different reading could also be proposed. The realization of pressure ulcers in areas where such lesions do not usually occur could indicate a particularly important weight of the endogenous predisposing characteristics of the patient, and therefore lead to the conclusion that the subject is so predisposed that he/she develops ulcers even in uncommon sites.

## 6. Conclusions

The nearly equivalent contribution of endogenous and exogenous elements in the development of decubitus injuries makes it an issue of intense medico-legal interest. In fact, it is on the concept of “preventability” that the entire discussion focuses with respect to identifying health-related professional liability profiles. Medical advances and ever-increasing patient expectations move the expected level of preventability ever higher. This is also because international scientific societies are drawing up increasingly accurate and precise guidelines, and the application of which should theoretically reduce the risk of developing pressure ulcers almost to zero. However, it should also be considered that the more articulated and complex the guidelines are, the greater the difficulty in applying them in practice, especially in settings where there are many critically ill patients. The role of the adequate documentation of prevention activities is undoubtedly crucial, as is the importance of constant updating by healthcare personnel.

Based on the review of the literature and the case presented, it is reasonable to conclude that the tools available today are certainly able to significantly reduce the risk of the development of pressure ulcers, but that there is still a certain proportion of injuries (difficult to quantify) whose development is independent of the adequacy of health care conduct. In such cases, liability profiles can only be excluded through the demonstration of an impeccable prevention policy, and this demonstration can only be based on the examination of clinical documentation, in which all the guidelines and protocols followed must be detailed.

## Figures and Tables

**Table 1:** SANRA Score for quality assessment of selected studies for the review.

Reference and Year of Publication	Justification of the Article’s Importance for the Readership	Statement of Concrete Aims or Formulation of Questions	Description of the Literature Search	Referencing	Scientific Reasoning	Appropriate Presentation of Data	Total Score
Lockhart [14]	2	2	1	1	1	1	8
Meehan & Hill [15] 2002	2	1	1	1	1	1	7
Nelson [16] 2003	2	2	1	2	1	1	9
Dimond [17] 2003	2	1	1	1	1	1	7
Heinemann et al. [18] 2003	2	2	1	1	1	1	8
Bergstrom [19] 2005	1	2	1	1	1	1	7
Lyder [20] 2005	2	2	2	2	2	1	11
Clarkson [21] 2007	1	2	1	1	1	1	7
Ayello et al. [22] 2009	2	2	2	2	2	2	12
Guy [23] 2010	1	2	1	1	1	1	7
Lyder [24] 2011	2	1	1	1	1	1	7
Genesio [25] 2016	1	1	1	1	1	1	6

**Table 2:** Summary of the content of the 12 articles included in the review.

Reference and Year of Publication	Socio-Environmental Context	Type of Article	Title	Framing of Liability Profiles for Healthcare Professionals	Proposals to Contain Litigation
Lockhart [14] 2002	USA	Perspective Article	The legal implications of pressure ulcers in acute care	The element that most often makes it impossible for the health care professional to defend himself/herself in court is the lack of documentation of preventive actions in the medical record	Implementing customized preventive measures Knowing and adopting care protocols Documenting any medical action performed (also the unsuccessful attempts to implement preventive measures)
Meehan & Hill [15] 2002	USA	Perspective Article	Pressure ulcers in nursing homes: does negligence litigation exceed available evidence?	Medico-legal litigation is driven by a lack of knowledge about aging and its consequences, leading to unrealistic expectations about functional improvements for family members in nursing homes	Involving family members as partners in care Documenting pressure ulcer-related factors in the context of the individual patient (rather than focusing on the wound physical characteristics) Ensuring that nursing home providers, caregivers, and community groups play an active role in demanding that funding sources be directed to the pressure sores issue
Nelson [16] 2003	Australia	Perspective Article	Pressure ulcers in Australia: patterns of litigation and risk management issues	Health care users are increasingly aware of medical advances and therefore require that standards of care be very high	Adhering to updated clinical practice guidelines for pressure ulcers and standards for wound management Establishing a climate of good communication among members of the health care team Making sure the medical record is up-to-date, clear, and easy to read
Dimond [17] 2003	United Kingdom	Perspective Article	Pressure ulcers and litigation	The realization or aggravation of pressure sores is no longer seen as an unavoidable consequence of a chronic disease condition or bedding, but as evidence of inadequate health care	High standards of documentation Continuing education of healthcare professionals Awareness that the applicability of guidelines must be assessed on a case-by-case basis
Heinemann et al. [18] 2003	Germany	Research Article	Medico-legal aspects of pressure sores	There is a consensus that decubitus injuries can be prevented through standardized nursing schemes that include mobilization, control of nutrient and fluid intake, and bedding programs, but it is still not well understood to what extent this is true	Classification of pressure sores according to standardized scales (Norton, Braden, Waterlow) Promotion of large-scale care quality improvement programs managed by nursing professionals
Bergstrom [19] 2005	USA	Editorial	Litigation or redesign: improving pressure ulcer prevention	Juries tend to side with plaintiffs, awarding higher damages when more risk factors are present	Fostering cooperation among clinicians and institutions Sharing information between patients and providers Adopting evidence-based decisions
Lyder [20] 2005	USA	Perspective Article	Medico-legal implications (chapter of the book “Pressure Ulcer Research”)	The ever-increasing knowledge level of the general public significantly raises the bar in terms of quality of care	Increasing regulations related to pressure ulcer care Emphasizing the economic repercussions of litigation related to bedsores so that health care administrations always take the issue into the highest consideration
Clarkson [21] 2007	United Kingdom	Review Article	Are pressure ulcers an act of nursing negligence?	There is growing awareness that through the adoption of appropriate control and prevention measures, pressure sores are in most cases preventable	Documenting and keeping proper medical record Following clinical guidelines Keeping up to date on advances in care
Ayello et al. [22] 2009	USA	Consensus Paper	Legal issues in the care of pressure ulcer patients: key concepts for health care providers: a consensus paper from the international expert wound care advisory panel	The average age of the population is increasing, making the delivery of effective, quality healthcare very complex. Nonetheless, citizens want standards of care to remain high.	Describing the injuries very accurately Scrupulously documenting the interventions taken and the patient’s responses to those interventions Carefully discussing “unavoidable” pressure sores in the patient record
Guy [23] 2010	United Kingdom	Perspective Article	Accountability and legal issues in tissue viability nursing	On the one hand there is poor communication between doctor and patient, and on the other hand there is often lack of understanding on the part of the patient	Accurately documenting all measures taken to prevent the occurrence of bedsores Gaining an in-depth understanding of pressure sore management
Lyder [24] 2011	USA	Perspective Article	Preventing heel pressure ulcers: economic and legal implications	It is generally assumed that the occurrence of pressure ulcers is due to negligence on the part of physicians or nurses	Conducting as accurate a risk assessment as possible Carefully documenting all preventive measures implemented Adopting heel protectors
Genesio [25] 2016	USA	Perspective Article	Pressure Ulcers Are Easy Pickings for Lawsuits Provider	The susceptibility of some patients to the development of pressure sores should dictate the closest monitoring possible	Employing a specific wound care policy detailing the responsibilities for each staff member involved in the process Regularly training medical and nursing staff in the care and prevention of pressure sores

## Data Availability

Not applicable.
